# Therapeutic effect of qinghuanling on negative symptoms and cognitive function of schizophrenia

**DOI:** 10.1192/j.eurpsy.2021.2048

**Published:** 2021-08-13

**Authors:** Y. Zhang, X. Cui

**Affiliations:** Pharmacy Lab, Xi’an Mental Health Center, Xi’an, China

**Keywords:** Quetiapine, Qinghuanling, Negative factor, Cognitive factor

## Abstract

**Introduction:**

Therapeutic effect of Qinghuanling on negative symptoms and cognitive function of schizophrenia

**Objectives:**

To evaluate the therapeutic effect of Qinghuanling on cognitive impairment in schizophrenia, and to provide basis for clinical medication.

**Methods:**

24 male patients with schizophrenia were randomly divided into study group and control group. The study group was given quetiapine fumarate combined with Qinghuanling, and the control group was given quetiapine fumarate. The positive and negative symptom scale (PANSS) and adverse event response scale (TESS) were evaluated regularly.

**Results:**

The PANSS score of the study group was significantly lower than the control group from 6th week (64.10 ± 7.64 vs 72.31 ± 11.16; 51.60 ± 7.40 vs 63.23 ± 7.08, P < 0.05). Among them, the score of negative factor in the study group was significantly lower than that in the control group at the end of 6 and 8 weeks (2.16 ± 0.40 vs 2.75 ± 0.38; 1.65 ± 0.42 vs 2.38 ± 0.43, P < 0.01); the score of cognitive factor in the study group was significantly lower than that in the control group at the end of the 8th week (1.87 ± 0.20 vs 2.12 ± 0.27, P < 0.05). Compared with before treatment, PANSS score and symptom cluster factor score of the two groups were significantly decreased from the 2nd weekend to the 8th weekend (P < 0.05).

**Conclusions:**

The combined use of Qinghuanling can significantly improve the therapeutic effect of schizophrenia, especially for the symptom cluster score of negative factors and cognitive factors, with high safety.

**Disclosure:**

No significant relationships.

